# Duplex ultrasound assessment after venous vascular closure of 16–17 F OD femoral venotomy: AMBULATE EXPAND ultrasound substudy

**DOI:** 10.1016/j.hroo.2026.03.018

**Published:** 2026-04-23

**Authors:** T. Jared Bunch, Suneet Mittal, Amin Al Ahmad, Maheer Gandhavadi, Zayd A. Eldadah, David De Lurgio, Bruce Hook, Joseph Banno, Hugh T. McElderry

**Affiliations:** 1 University of Utah Hospital, Salt Lake City, Utah; 2 Electrophysiology, Valley Health System, Paramus, New Jersey; 3 Texas Cardiac Arrhythmia Institute, St. David’s Medical Center, Austin, Texas; 4 Overlake Medical Center, Bellevue, Washington; 5 Heart & Vascular Institute, MedStar Health, Columbia, Maryland; 6 Emory Healthcare, Atlanta, Georgia; 7 Department of Cardiology, Lahey Hospital & Medical Center, Burlington, Massachusetts; 8 Department of Medicine, University of Alabama at Birmingham, Birmingham, Alabama

**Keywords:** Atrial fibrillation ablation, Left atrial appendage closure, Anticoagulation, Venous closure, Duplex ultrasound, Electrophysiology

**Key Findings**

▪ In a core laboratory–adjudicated duplex ultrasound (DUS) substudy of 29 evaluable patients after VASCADE MVP XL closure of 16–17 F OD femoral venotomy, 89.7% showed no abnormal DUS findings, and no subject required intervention for detected abnormalities.
▪ 3 abnormal findings (10.3%) were detected (2 partially occlusive common femoral vein thromboses [6.9%] and 1 hematoma [3.4%]), all asymptomatic in a high-risk, fully anticoagulated cohort.
▪ Early postprocedure DUS confirmed preserved venous compressibility and respiratory phasicity in the vast majority, supporting that the device maintains early venous integrity and aligns with low complication rates reported for other large-bore venous closure strategies.

The rapid evolution and expansion of complex electrophysiology and structural heart interventions have necessitated routine use of large-bore (16–24 F) femoral venous access. As these procedures are increasingly performed with a goal of same-day discharge, early hemostasis and rapid return to ambulation have become critical operational metrics. Within this modern care paradigm, tools that minimize postprocedural management, improve predictability from admission through discharge, and facilitate early ambulation while preserving patient comfort are critical.

The VASCADE MVP Venous Vascular Closure System was developed to address these needs by delivering a resorbable extravascular collagen patch to the tissue tract (Figure 1). The pivotal AMBULATE trial demonstrated safety and efficacy of VASCADE MVP for 6–12 F inner diameter [15 F outer diameter (OD)] sheaths, reducing time to ambulation and discharge.^1^ The subsequent AMBULATE EXPAND trial evaluated the MVP XL system for larger sheaths (16–17 F OD); in 77 subjects, the device demonstrated 98.7% success and 0% major complications, with a mean time to hemostasis of 2.7 minutes and ambulation in 3.2 hours.^2^ However, clinical surveillance alone relies on palpable or visible signs of complications and may underestimate subclinical vascular sequelae, such as nonocclusive thromboses, small pseudoaneurysms, or arteriovenous (AV) fistulas. To rigorously evaluate the anatomical integrity of the femoral vein after collagen-based closure of large-bore access, objective imaging is required. This substudy used standardized core laboratory–adjudicated duplex ultrasound (DUS) to characterize early local vascular effects in this high-risk population.

**Figure 1:**
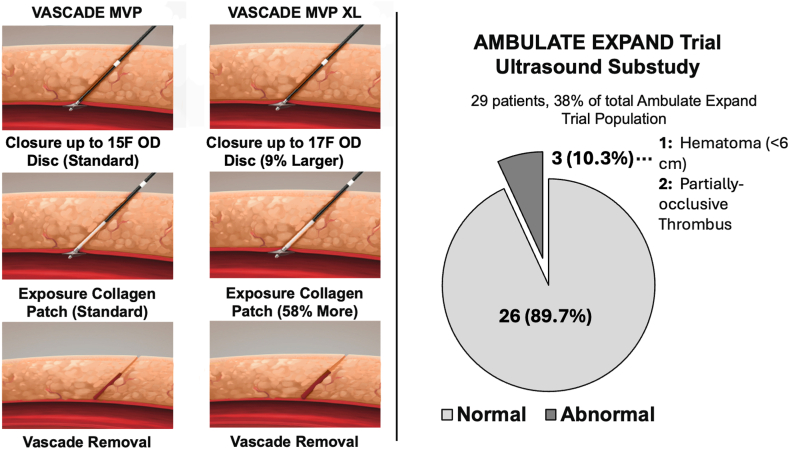
*Left:* Comparison of the VASCADE MVP and VASCADE MVP XL venous closure systems. *Right:* Summary of the duplex ultrasound findings for all patients in the substudy.

5 sites prospectively enrolled substudy subjects until ≥25 cases with evaluable images were available. The research reported in this article adhered to the Declaration of Helsinki and relevant institutional guidelines; the study protocol was approved by all enrolling institutional review boards; and all participants provided written informed consent. DUS of the study access site was performed following a predefined core laboratory imaging manual. Scans were to be conducted between documented successful ambulation and 5 days postprocedure. DICOM images and site worksheets were uploaded to Syntropic Core Lab for review.

Core laboratory end points included compressibility and respiratory phasicity of the common femoral vein (CFV), vessel diameter, and the presence of thrombosis, hematoma, pseudoaneurysm, AV fistula, and stenosis. Findings were communicated to site investigators for adverse event adjudication.

31 subjects consented to the substudy (29 completed DUS with evaluable images and comprised the analytical cohort). Subjects were predominantly male (23, 79.3%), with a mean age of 67.8 ± 10.9 years. The population displayed a high prevalence of metabolic risk factors, including a mean body mass index of 30.3 ± 5.5 kg/m^2^, hypertension (18, 62.1%), and hypercholesterolemia (19, 65.5%). Notably, 29 (100%) of subjects were taking oral anticoagulant/antiplatelet medications at the time of the procedure. The procedures performed were cardiac ablation (22, 75.9%) for atrial fibrillation (AF) with pulsed field ablation, AF with pulsed field ablation plus left atrial appendage closure (4, 13.8%), and left atrial appendage closure alone (3, 10.3%).

Most ultrasound examinations were performed after ambulation but before discharge (77.4%), with 4 examinations completed within 5 days of discharge. Core laboratory reads showed no abnormal DUS findings in 26 of 29 subjects (89.7%) (Figure 1). An abnormal finding was present in 3 of 29 subjects (10.3%), of whom, only 2 had site-reported adverse events (6.9%). Visible partially occlusive CFV thrombosis was noted in 2 of 29 (6.9%), and a hematoma was noted in 1 of 29 (3.4%)—4.2 cm widest diameter. CFV compressibility was abnormal in the thrombosis cases, though respiratory variation was normal in all subjects. No stenosis was identified. Neither AV fistula nor pseudoaneurysm was identified in 28 subjects; 1 subject’s examination lacked arterial imaging and was indeterminate for those specific end points. Importantly, no subject required intervention for core laboratory–detected abnormalities.

Core laboratory–adjudicated findings confirmed preserved early venous integrity in the majority of patients after VASCADE MVP XL closure of 16–17 F OD femoral venotomy. Despite this high-risk cohort (obesity and universal anticoagulation), only 2 partially occlusive thromboses and hematoma (all asymptomatic) were observed.

This profile is consistent with contemporary reports of device- and suture-mediated large-bore venous closure. Prior AMBULATE data and other case series have shown that device-based venous closure reduces time to hemostasis and ambulation, with low overall clinically apparent complication rates.^1^ In patients undergoing structural heart and electrophysiology procedures, use of preclosure suture techniques and dedicated venous closure devices has yielded low clinically significant hematoma and thrombotic complication rates with large-bore venous access sheaths.^3^^,^^4^

Comparative imaging studies demonstrate that routine postprocedural DUS detects more subclinical abnormalities than clinical surveillance alone. Earlier DUS (≤5 days from the procedure) may be more sensitive to detecting abnormalities than later DUS used in prior trials. However, the clinical relevance of these additional findings may be limited. In cohorts with systematic DUS after AF ablation with smaller venous access sheaths, the reported imaging-detected hematoma rate was 3.5% and the overall DUS-detected vascular complication rate was 6.4%.^5^ The single hematoma (3.4%) and partially occlusive thromboses (6.9%) observed here align with these reported ranges, despite insertion of larger venous sheaths. Importantly, the absence of pseudoaneurysm, AV fistula, or stenosis in our cohort is significant, as these complications often require intervention.^5^

From a mechanistic standpoint, retention of respiratory phasicity and vessel compressibility indicates that venous hemodynamics remain intact. Preserving this functional integrity is key to mitigating the risk of long-term venous sequelae. The instances of partially occlusive CFV thromboses detected by DUS appear owing to localized stasis or endothelial trauma associated with large-bore access, rather than intrinsic device failure.

In conclusion, centrally adjudicated DUS after VASCADE MVP XL closure of 16–17 F OD femoral venotomy demonstrated predominantly normal early vascular findings in a high-risk, fully anticoagulated population. Addressing the current scarcity of objective imaging data in large-bore venous closure, these results provide reassuring evidence that the device preserves vessel integrity and hemodynamics, effectively mitigating thrombotic and bleeding risks across a variety of cardiovascular procedures.

## References

[1] Natale A., Mohanty S., Liu P.Y. (2020). Venous vascular closure system versus manual compression following multiple access electrophysiology procedures: the AMBULATE trial. JACC Clin Electrophysiol.

[2] McElderry H.T., Al-Ahmad A., De Lurgio D.B. (2026). Safety and efficacy of the VASCADE MVP® XL Venous Vascular Closure System for management of femoral venotomy following catheter-based interventions utilizing 16–17F OD sheaths: the AMBULATE EXPAND trial. J Cardiovasc Electrophysiol.

[3] Ali M., Masood F., Erickson L. (2024). Suture closure AFtEr large bore vein access (SAFE-VEIN): a randomized, prospective study of the efficacy and safety of venous closure device. Catheter Cardiovasc Interv.

[4] Mohammed M., Nona P., Abou Asala E. (2022). Preclosure of large bore venous access sites in patients undergoing transcatheter mitral replacement and repair. Catheter Cardiovasc Interv.

[5] Lee H.J., Lee S.H., Park S. (2025). Incidence, predictors, and management of femoral vascular complications following catheter ablation for atrial fibrillation: a systematic duplex ultrasound study. Biomedicines.

